# Real-Time Jellyfish Classification and Detection Based on Improved YOLOv3 Algorithm

**DOI:** 10.3390/s21238160

**Published:** 2021-12-06

**Authors:** Meijing Gao, Yang Bai, Zhilong Li, Shiyu Li, Bozhi Zhang, Qiuyue Chang

**Affiliations:** 1 College of Information and Electronic, Beijing Institute of Technology, Beijing 100081, China; 2 The Key Laboratory for Special Fiber and Fiber Sensor of Hebei Province, School of Information Science and Engineering, Yanshan University, Qinhuangdao 066004, China; baiyang@stumail.ysu.edu.cn (Y.B.); zhilongli4212@162.com (Z.L.); milanlsy@126.com (S.L.); zhangbozhi93@126.com (B.Z.); qiuyuechang@126.com (Q.C.)

**Keywords:** jellyfish, convolutional neural network, image processing, YOLOv3

## Abstract

In recent years, jellyfish outbreaks have frequently occurred in offshore areas worldwide, posing a significant threat to the marine fishery, tourism, coastal industry, and personal safety. Effective monitoring of jellyfish is a vital method to solve the above problems. However, the optical detection method for jellyfish is still in the primary stage. Therefore, this paper studies a jellyfish detection method based on convolution neural network theory and digital image processing technology. This paper studies the underwater image preprocessing algorithm because the quality of underwater images directly affects the detection results. The results show that the image quality is better after applying the three algorithms namely prior defogging, adaptive histogram equalization, and multi-scale retinal enhancement, which is more conducive to detection. We establish a data set containing seven species of jellyfishes and fish. A total of 2141 images are included in the data set. The YOLOv3 algorithm is used to detect jellyfish, and its feature extraction network Darknet53 is optimized to ensure it is conducted in real-time. In addition, we introduce label smoothing and cosine annealing learning rate methods during the training process. The experimental results show that the improved algorithms improve the detection accuracy of jellyfish on the premise of ensuring the detection speed. This paper lays a foundation for the construction of an underwater jellyfish optical imaging real-time monitoring system.

## 1. Introduction

The ocean covers about 71% of the total area of the earth. Although human beings have abundant resources, exploration and development are still very limited. With the development of science and technology, marine resources have attracted increasing attention. In recent years, deep-sea information technology has developed rapidly, and the effective utilization of marine resources plays an important role in China’s national economy and national defense construction, which is very important for further developing marine resources and collecting, transmitting, and processing marine information [1].

Under the influence of climate change and the intensification of human activities, anomalies have appeared in the marine system. Large-scale jellyfish outbreaks have occurred in China’s offshore waters, causing severe ecological damage and economic losses [2].

The coastline of Hebei Province is about 500 km long. In recent years, jellyfish outbreaks and other disasters have occurred in the sea area of Hebei Province, which endangers the safety of people and fisheries. These are major problems that affect the development of tourism, fishing, and the marine ecological balance in Hebei Province. Over the last few years, ecological disasters frequently broke out in the Qinhuangdao Sea area [3]. Primarily, the mass reproduction of jellyfish has led to frequent jellyfish outbreaks in many sea areas, which has caused severe losses to the local ecology and economy. For example, jellyfish sting tourists and threaten their lives, and excessive reproduction breaks the ocean’s ecological balance [4]. These seriously affected the coastal environmental security and the sustainable development of the marine economy. Therefore, accurate monitoring of jellyfish species, distribution, and quantity is crucial [5]. However, due to the complex marine environment and immature related technologies, the research on underwater monitoring and early warning technology of jellyfish has become a research hotspot and difficulty.

The underwater monitoring process of jellyfish includes underwater imaging and image monitoring. At present, the maturest technologies of underwater imaging are acoustic imaging and optical imaging. Acoustic imaging monitors jellyfish by obtaining the internal structure of objects by sound waves, while optical imaging monitors jellyfish by transmitting and receiving optical signals. Compared with acoustic imaging, optical imaging has a higher resolution, which makes optical imaging develop rapidly in underwater target detection. Therefore, the underwater optical imaging method can be used to image the jellyfish, and then the optical image of the jellyfish can be detected later.

A convolutional neural network (CNN) applied to the detection algorithm is a new target monitoring method at present. Unlike traditional methods, it can adaptively learn the characteristics of input data through a neural network and make the next prediction. Therefore, this method has gradually developed into a new monitoring method in marine life research, including fish, shrimp, and jellyfish. In this paper, the algorithm of jellyfish detection based on CNN is studied using CNN and image processing theory.

The CNN-based target detection algorithm is mainly divided into two foundations: based on region recommendation, such as Faster R-CNN, and based on regression, such as YOLOv3, according to the principle. Because Faster R-CNN has the problem of low real-time, this paper uses the YOLOv3 algorithm to detect jellyfish, which improves the real-time performance of jellyfish detection. However, the YOLOv3 algorithm has the problem of good real-time performance but low accuracy. Therefore, on the premise of ensuring real-time, it is necessary to optimize the feature extraction network of YOLOv3-Darknet53: specifically, add large-step cross-layer residual connections to the Darknet53 network to improve the feature extraction capability of Darknet53. A top-down pyramid structure is added to the feature pyramid, and the information of the feature map is repeatedly extracted, thereby improving the detection accuracy. The learning rate method of label smoothing and cosine annealing learning rate is introduced in the training process to improve network accuracy.

## 2. Related Work and Contributions

This section introduces the existing work related to the detection and monitoring of jellyfish and then presents the main contributions of this paper.

### 2.1. State-of-the-Art

The traditional method of detecting plankton is using trawl, but this approach has low efficiency. With the development of science and technology, researchers use various methods to detect underwater creatures, such as optical imaging, sonar imaging, remote sensing sensors, etc.

Houghton et al. applied aerial photography technology to the distribution and movement characteristics of jellyfish. The method draws researchers’ attention to the wide application of aerial surveys in the study of gelatinous aggregates beyond the scope of traditional shipborne technology. However, due to the incapability of collecting comparable data on the relative abundance among species, this approach cannot identify jellyfish underlying the water surface [6].

Some researchers have used sonar and hyperspectral remote sensing technology to image harmful jellyfishes. Gustavo Alvarez Colombo et al. found that jellyfish contribute significantly to the sound scattering even at pretty low frequencies. Therefore, the team used sonar detectors to detect jellyfish individuals throughout the waters, but this method cannot be used to monitor tiny jellyfish [7].

Wang Jianyan et al. established a preliminary molecular biological identification method and detection technology of jellyfish, which got rid of the limitations of jellyfish’s current morphological identification method and could accurately and quickly qualitatively analyze samples, but it was limited to qualitative and semi-quantitative research of jellyfish [8].

Hangeun Kim et al. put forward an unmanned jellyfish monitoring system, which used unmanned aerial vehicles to observe jellyfish on the sea surface while flying and recognized the jellyfish through deep learning, realizing accurate recognition of jellyfish. However, this method can not detect jellyfish in the deep sea [9].

Seonghun Kim et al. investigated the spatial and vertical distribution of jellyfish by acoustic and optical methods, but this method had limitations in monitoring tiny jellyfish [10].

Donghoon Kim et al. designed an autonomous jellyfish cleaning robot system, which can monitor jellyfish, with a monitoring rate of over 99%, but it cannot effectively classify jellyfish species [11].

Jungmo Koo et al. designed a management system of jellyfish distribution using an unmanned aerial vehicle and an unmanned surface vehicle. A deep neural network structure with high precision and fast operation speed was proposed to accurately identify the target jellyfish. However, this method can only identify a single species [12].

French et al. monitored jellyfish through underwater imaging equipment and used a multistage neural network classifier to identify them. The experimental results showed that the accuracy of correct classification was as high as 90%, indicating that the system can effectively evaluate jellyfish outbreaks. Still, the system cannot detect numbers of individual jellyfish outbreaks [13].

Martin Vodopiveca et al. developed a method to detect and calculate numbers of jellyfish hydra larvae automatically. This method can monitor numbers of jellyfish larvae, evaluate the quality of manual counting, and solve the problem of huge differences between different annotators’ comments [14]. The proposed algorithm proves that jellyfish larvae can be identified by optical imaging and automatic image. However, it is still in an off-line recognition state and cannot be monitored in real-time.

Martin-Abadal et al. put forward the Jellytoring system, which used a deep object detection neural network to detect and quantify different kinds of jellyfish automatically and can automatically record the existence of jellyfish for a long time. Still, the applicability of this system is limited [15].

In the past, researchers mainly realized jellyfish detection by combining acoustic imaging, optical imaging, image processing technology, and deep learning methods. However, the research on jellyfish is still in the primary stage and the existing methods are not systematic enough, so a few jellyfish species can be identified. Deep learning technology is gradually applied to jellyfish detection because of its high efficiency, rapidity, and high accuracy. Therefore, based on CNN theory and digital image processing technology, seven species of jellyfishes and fish are detected. This study lays a theoretical and technical foundation for constructing an underwater jellyfish optical imaging system.

### 2.2. Contributions

The main contributions of this paper are as follows:

(1) A data set including seven types of jellyfishes and fish are established in this paper, including *Cyanea purpurea*, *Rhizostoma pulmo*, *Phacellophora camtschatica*, *Agalma okeni*, *Aurelia aurita*, *Phyllorhiza punctata*, *Rhopilema esculentum*, and fish. A total of 2141 images are included in the data set.
(2) In this paper, the jellyfish detection algorithm is based on the YOLOv3 target detection algorithm. The Darknet53 feature extraction network is optimized by introducing a large-step cross-layer residual connection and a top-down pyramid structure.
(3) When training the improved network, two training methods, label smoothing, and a cosine annealing learning rate are introduced to improve the overall detection effect of the algorithm.
(4) This paper provides a theoretical and technical basis for the subsequent realization of jellyfish real-time monitoring.

The remainder of this paper is organized as follows. Section 2 reviews related work on jellyfish detection. Section 3 describes data set preprocessing and preparation. In Section 4, we present the classification of jellyfish based on the improved YOLOv3. We illustrate the experimental process and results in Section 5. Finally, in Section 6, we summarize the paper, list our methods’ advantages, and point out the following research direction.

## 3. Data set Preparation and Preprocessing

### 3.1. Data Set Preparation

**Figure 1:**
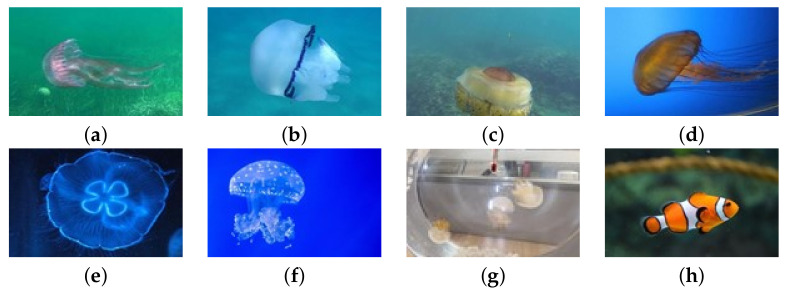
The example of the target sample. (**a**) *C. purpurea*; (**b**) *R. pulmo*; (**c**) *P. camtschatica*; (**d**) *A. okeni*; (**e**) *A. aurita*; (**f**) *P. punctata*; (**g**) *R. esculentum*; (**h**) fish.

This paper is to test jellyfish, but there is only one published jellyfish data set [15]. Therefore, some images in this data set were obtained on the open data set website by crawler technology, and some images were collected by laboratory jellyfish. According to biological taxonomy, the creatures in the picture can be divided into eight categories, including seven types of jellyfishes and fish, which are *Cyanea purpurea*, *Rhizostoma pulmo*, *Phacellophora camtschatica*, *Agalma okeni*, *Aurelia aurita*, *Phyllorhiza punctata*, *Rhopilema esculentum*, and fish. Among them, there are many kinds of fishes, but there is no specific distinction between species. The data set of *C. purpurea*, *R. pulmo*, and *P. camtschatica* come from the data set published by Miguel Martin-Abadal et al. The data set of *A. okeni*, *A. aurita*, *P. punctata*, and fish comes from the network and is obtained by reptiles. The data set of *R. esculentum* is collected by us in the laboratory. A total of 2141 images are included in the data set, and the image resolution is roughly 500 × 350 pixels. Figure 1 shows the selected target samples to be identified. The number of each species of jellyfish and fish are shown in Table 1.

According to the requirements of later experiments, the data set is divided into the training set, the verification set, and the test set according to the ratio of 8:1:1. Table 2 shows the setting of the training set, the verification set, and the test set in the data set.

### 3.2. Underwater Image Preprocessing

**Figure 2:**
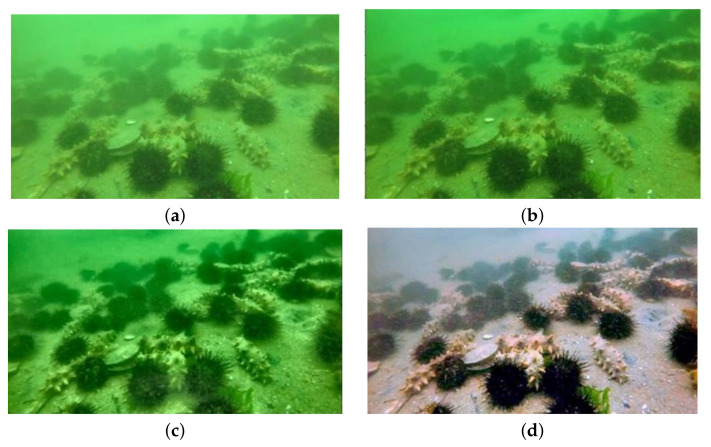
The results of different algorithms’ underwater image enhancement: (**a**) original image; (**b**) dark channel prior defogging; (**c**) histogram equalization processing; (**d**) multi-scale retinal enhancement with color recovery.

The absorption and scattering of light by seawater leads to blurred details, low contrast, and color distortion of underwater images [10]. Therefore, it is necessary to preprocess underwater images to improve the image quality. In this paper, we use three algorithms, namely prior defogging [16,17,18], adaptive histogram equalization [19,20], and multi-scale retinal enhancement [21,22], to process the original underwater image. The processing result is shown in Figure 2.

It can be seen from Figure 2 that the image defogging effect after dark channel before defogging is remarkable, with stronger contrast and richer colors. However, the visual effect of the whole image after processing is greener. When using CNN for target detection, the color of the target is an important feature, so it is important to restore the detected color to the color of the target itself. Although the whole image after the histogram equalization is still green, the visual effect is better than that of the dark channel prior to the defogging method. This method makes the brightness more evenly distributed on the histogram, and the whole processing process is intuitive and straightforward with less computation. However, as it does not screen data, the result may enhance the contrast of background noise and reduce the contrast of effective information. The image processed by the multi-scale retinal enhancement algorithm has an excellent visual effect, and the overall effect is better than the previous two methods. The brightness and color saturation of the image are restored, the color is clear, which is more in line with the visual characteristics, and the color of the object itself is restored to a great extent. It can be seen from the visual effect that the image processed by the multi-scale retinal enhancement algorithm is clearer, less noisy, and contains more information than the other two methods.

The corresponding evaluation parameters are shown in Table 3. The bold data in Table 3 indicate the optimal value of each parameter.

The simulation and experimental results show that the brightness and color saturation of the image processed by the multi-scale retinal enhancement algorithm are restored, and the SNT, PSNR, and SSIM values are better than the other two methods, and the visual effect is the best. We will build a polarized underwater imaging system in future work. The collected images are blurred due to poor water quality, so we will adopt the multi-scale retinal enhancement algorithm as the next underwater image preprocessing method.

## 4. Jellyfish Detection Based on Improved YOLOv3

The YOLO algorithm creatively combines the two stages of the candidate region and target recognition into one, which accelerates the detection speed and has been widely used in the industry. There are three main algorithms in this series: YOLOv1 [23], YOLOv2 [24], and YOLOv3 [25]. YOLOv3 is widely used in detection tasks. YOLO series algorithm is a one-stage algorithm, while Faster R-CNN is a two-stage algorithm. In principle, the one-stage algorithm is faster, but its accuracy is not as good as the two-stage algorithm. In other words, YOLOv3 is to reduce the precision value in exchange for improving the detection speed. Therefore, to ensure the real-time performance of the YOLOv3 algorithm, this paper will adopt two methods to improve the accuracy of the YOLOv3 algorithm and make it meet the requirements of jellyfish detection accuracy and speed. Specific improvements are as follows: (1) Optimize the Darknet53 feature extraction network of YOLOv3. The optimization method is to introduce a large-step cross-layer residual connection and add a top-down pyramid structure. (2) When training the improved network, two training methods, label smoothing, and the cosine annealing learning rate are introduced to improve the overall detection effect of the algorithm.

### 4.1. Improvement of Feature Extraction Network

(1) Large-step cross-layer residual connection method

The feature extraction network of YOLOv3, Darknet53, is composed of a series of residual structures. The operation of each residual structure is as follows: perform a 3 × 3 convolution on the input layer1 with a step size of 2, and then save the convolution result layer1, perform 1 × 1, 3 × 3 convolutions again, and add the convolution result into layer2 as the final result. The residual structure of the Darknet53 network is composed of repeated stacking according to the specified times (1, 2, 4, 6, 8).

According to the residual network theory, it is easy to optimize the network when using the residual structure, and the residual structure can effectively reduce the gradient explosion. Based on this, this paper introduces an improved large-step cross-layer residual connection structure. The specific structure is as follows: the residual blocks in the original YOLOv3 are grouped: one group processes the feature map accordingly to the original Darknet53 structure, and the other group starts from the first layer of the current stack structure, and the feature maps are directly connected to the last layer of the current stack structure after only a small amount of processing (convolution, normalization, activation function) [26].

**Figure 3:**
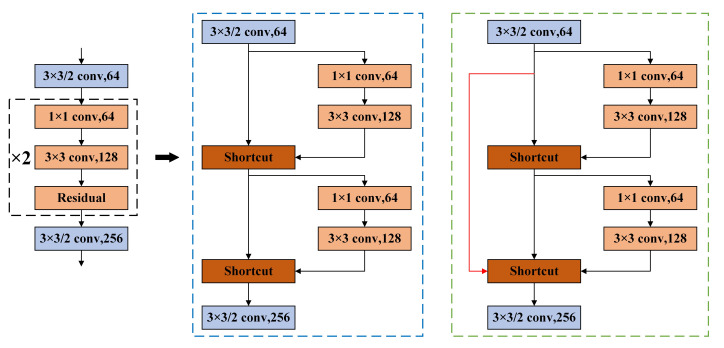
The improved residual structure.

Take the residual block stacked twice as an example, the network structure before and after improvement is shown in Figure 3. The black dotted line shows the schematic diagram of the residual block stacked twice, the blue dotted line shows the original residual structure, and the green dotted line shows the improved residual structure. A long-step cross-layer residual connection is equivalent to adding a completely independent gradient propagation path in the process of back propagation, enhancing gradient information and the learning ability of convolution neural networks.

(2) Feature pyramid structure improvement method

The feature pyramid structure (FPN) used in YOLOv3 mainly up samples the small-scale feature map (13 × 13) twice to obtain the size of the upper feature map (26 × 26), and then splices two feature maps (26 × 26) with the same size. In the same way, if the feature map obtained from the previous splicing is down-sampled twice to match the feature map with the same size in the original output, and then the two feature maps are spliced, the information contained in the newly obtained feature map will not be reduced, and a feature map that has richer semantic information and location information can be obtained theoretically [27]. In this paper, the top-down sampling part is introduced into the FPN of YOLOv3 to enrich the features of the feature map. Specific operations are as follows:

Firstly, the feature map D1 (52 × 52) obtained by the network structure of Darknet53 in Figure 3 is downsampled to obtain the feature map C2 (26 × 26).

Secondly, the feature map C2 and the feature map B1 are spliced again, and a new feature map B2 (26 × 26) is obtained again, which has a better effect on the detection of medium targets.

Thirdly, the feature map B2 (26 × 26) is downsampled and spliced with the original network output (13 × 13) feature map to generate a new feature map A2 (13 × 13), which is suitable for detecting large targets.

**Figure 4:**
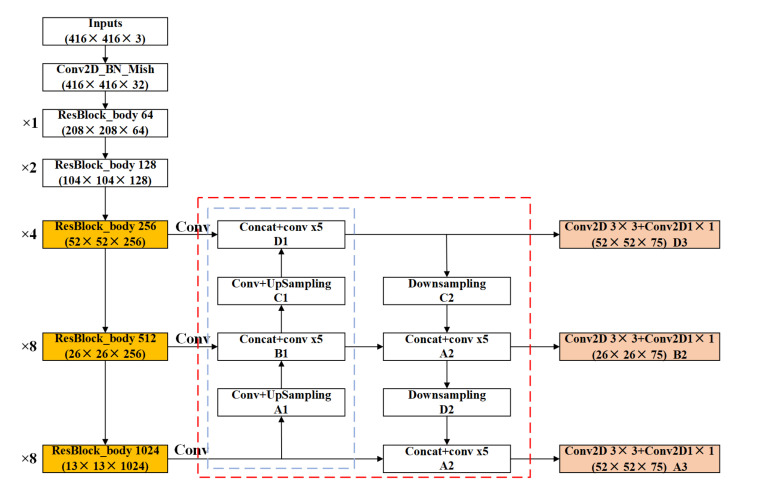
The network structure of the improved Darknet53.

The network structures with the improved FPN are shown in Figure 4. It can be seen that a very important feature of the FPN is to extract features repeatedly. In Figure 4, the blue dotted line is the traditional FPN, which completes feature extraction from the bottom to top. In contrast, the red dotted line is the improved pyramid structure, which includes both top-down feature extraction and down-sampling feature extraction from the bottom to top.

**Figure 5:**
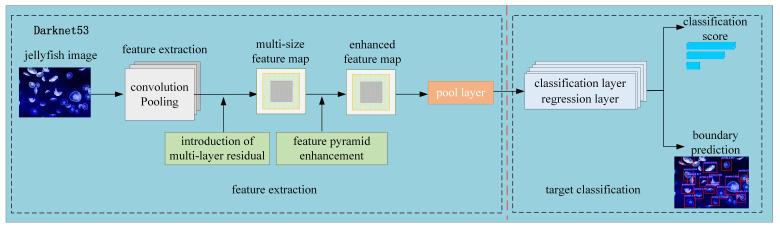
Schematic diagram of the improved YOLOv3.

To sum up, the schematic diagram of the improved YOLOv3 algorithm is shown in Figure 5. The features of the input jellyfish image are extracted by convolution, pooling, excitation function, etc. In this process, a multi-layer residual connection structure is introduced to output feature maps of three scales. After the improved FPN of the output feature map, the feature information is enhanced, and new feature maps with more information in three scales are generated. Then, the new feature map is sent to the subsequent pooling layer, classification layer, and regression layer. The jellyfish category (such as *A. aurita*, *R. esculentum*, etc.) in the boundary box is judged through the classification layer and the regression layer. The type’s confidence level is calculated. The position of the prior box is adjusted until it is adjusted to the target size, and the prediction of the target boundary box is completed.

### 4.2. Improvement of Training Method

To further improve the detection accuracy, a comprehensive algorithm is proposed in this paper. That is, the improved network is used for the feature extraction network of the algorithm, and the label smoothing method and cosine annealing learning rate method are introduced when training the network.

(1) Label smoothing method

When designing a classification task algorithm, it is generally considered that the probability of the target category in the label vector in training data is 1, and the probability of the non-target category should be 0. That is, the corresponding relationship between input and output is shown in Equation (1).

(1) $$f_{i}\;=\;\left\{\begin{array}{cc} 1, & i=\mathrm{target}\\ 0, & i\neq \mathrm{target} \end{array}\right.$$

Because there are many kinds of jellyfishes, and most of the individual shapes are umbrella caps and elongated tentacles, there are some similarities between individuals. The jellyfish data set is artificially labeled, which will inevitably lead to labeling errors. In the training process of the CNN, if there are wrong labels, the training results will be negatively affected. If the model can be "informed" that there may be errors in the labels, the trained model will be immune to a few standard wrong samples. This idea is called label smoothing [28]. Label smoothing refers to setting an error rate defined for the algorithm before network training, substituting ( $x_{i},1-y_{i}$ ) into training with the probability of σ and substituting ( $x_{i},y_{i}$ ) into training with the probability of ( 1−σ ) in each iterative training. At this time, it is equivalent to smoothing the labels. Suppose the smoothing coefficient is set to 0.01, and the original labels in the second classification task are 0 and 1. In that case, the labels can be changed to 0.01 and 0.99 after smoothing, which means that the classification accuracy is punished a little so that the model should not be classified too accurately. The probability of over-fitting can be reduced. Therefore, the improved algorithm in this chapter also introduces the label smoothing method to improve the accuracy. Considering that the probability of wrong labeling will not exceed 1%, the smoothing coefficient in this paper is set to 0.01.

(2) Cosine annealing learning rate

When using CNN to detect the target, because the optimization function of the target may be multimodal, if the gradient descent method is used to optimize the target function, the parameters may fall into the local minimum and swing around the local minimum, thus failing to reach the global optimum. When the objective function is approaching the global minimum, the learning rate should be reduced appropriately to avoid the loss value swinging around the global maximum but never reaching the maximum. The idea of a simulated annealing algorithm is introduced into learning rate attenuation to obtain the cosine annealing learning rate [29], which can effectively solve the above problems.

## 5. Experiment and Result Analysis

### 5.1. Experimental Process

(2) Experimental method

To verify the effectiveness of the improved network and training methods, this section will set up five groups of experiments. The five groups of experiments are: (1) original YOLOv3 algorithm; (2) improve the network structure; (3) Improve the network structure and use label smoothing; (4) improve the network structure and use cosine annealing learning rate; (5) comprehensively improved algorithm (including the improved network structure, label smoothing, and cosine annealing learning rate method).

The number of images in the training, the verification, and the test sets are 1713, 214, and 214, respectively. The intersection ratio threshold and confidence threshold of the algorithm are set to 0.5 and 0.5, respectively. Migration learning can save computing resources and improve network efficiency. Therefore, to reduce the training time and improve the network efficiency, the network initial parameter model adopts the parameters of the YOLOv3 model, and the parameters are mainly updated by the back propagation method in the training process. Then, the experimental results are analyzed by using the target detection evaluation parameters.

(2) Parameter setting

The super parameter settings during the experiment are shown in Table 4.

### 5.2. Data Analysis

In this paper, five evaluation indexes are used to analyze the experimental results quantitatively, and the results are as follows:

(1) Accuracy value analysis

**Figure 6:**
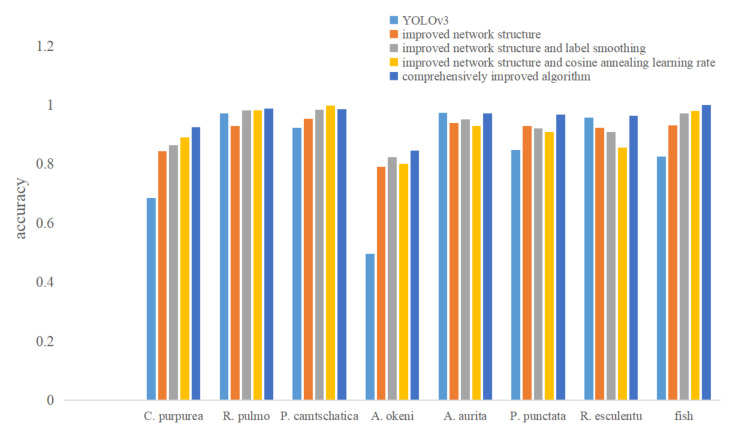
The summary of the five algorithms’ accuracy.

Table 5 is the accuracy result of the above five algorithms. Figure 6 summarizes the accuracy value result obtained in the five algorithms.

It can be seen from Table 5 that the detection accuracy of the comprehensively improved algorithm in this paper for almost all organisms is higher than that of the other four algorithms.

(2) Average accuracy analysis

Table 6 shows the comparison results of the mean Average Precision (mAP) of the above five algorithms, and the bold data are the mAP of the comprehensively improved algorithm. It can be concluded from Table 6:

The mAP of the algorithm with the improved network structure is higher than that of the original YOLOv3 algorithm. Both the label smoothing method and cosine annealing learning rate method can improve the mAP, and the label smoothing method can significantly improve the accuracy. The mAP of the comprehensively improved algorithm is 0.9553, which is better than the other four algorithms. The result indicates that the proposed comprehensively improved algorithm in this chapter has the highest detection accuracy.

In the preliminary work, our group used deep learning methods to build a Faster R-CNN detection algorithm based on the AlexNet network and the GoogLeNet network. The results show that the mAP of the two target detection algorithms are 0.5107 and 0.7496, respectively [30]. It is found that the mAP of the five algorithms proposed in this paper are higher than that of the previous algorithms by comparison. The results show that the algorithms can effectively improve classification accuracy.

(3)
$F_{1}$ -score analysis

**Figure 7:**
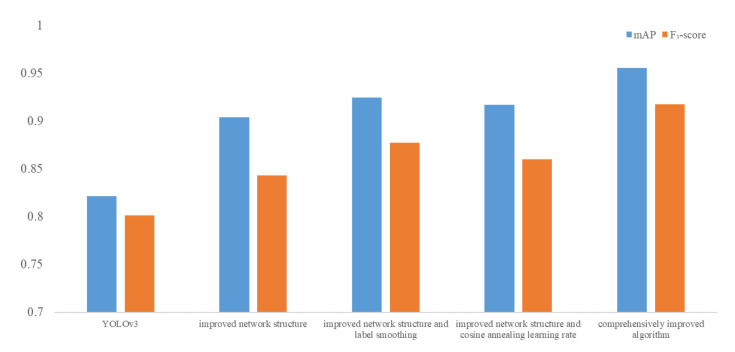
The summary of the five algorithms’ mAP and $F_{1}$ -score.

Table 7 shows the comparison results of the $F_{1}$ -score of the above five algorithms, and the bold data are the $F_{1}$ -score value of the comprehensively improved algorithm. The $F_{1}$ -score of the comprehensively improved algorithm is the largest. We can see from the $F_{1}$ -score that the comprehensively improved algorithm has the best effect. $F_{1}$ -score represents the ability of the model to balance precision and recall, and the larger the $F_{1}$ -score, the better the network performance. Figure 7 summarizes the results of the mAP and the $F_{1}$ -score obtained in the five algorithms.

(4) FPS analysis

Table 8 shows the comparison results of the FPS of the above five algorithms, and the bold data is FPS value of the comprehensively improved algorithm. We can see from Table 8 that the FPS value of the original YOLOv3 algorithm is the highest, but the other four algorithms are slightly lower than it, which is similar. That is, the detection speed of the five algorithms are equal.

From Table 6, Table 7 and Table 8, we can see that this paper comprehensively improves the algorithm, and the detection accuracy is significantly improved under the condition that the detection speed is basically unchanged.

(5) Loss function curve

**Figure 8:**
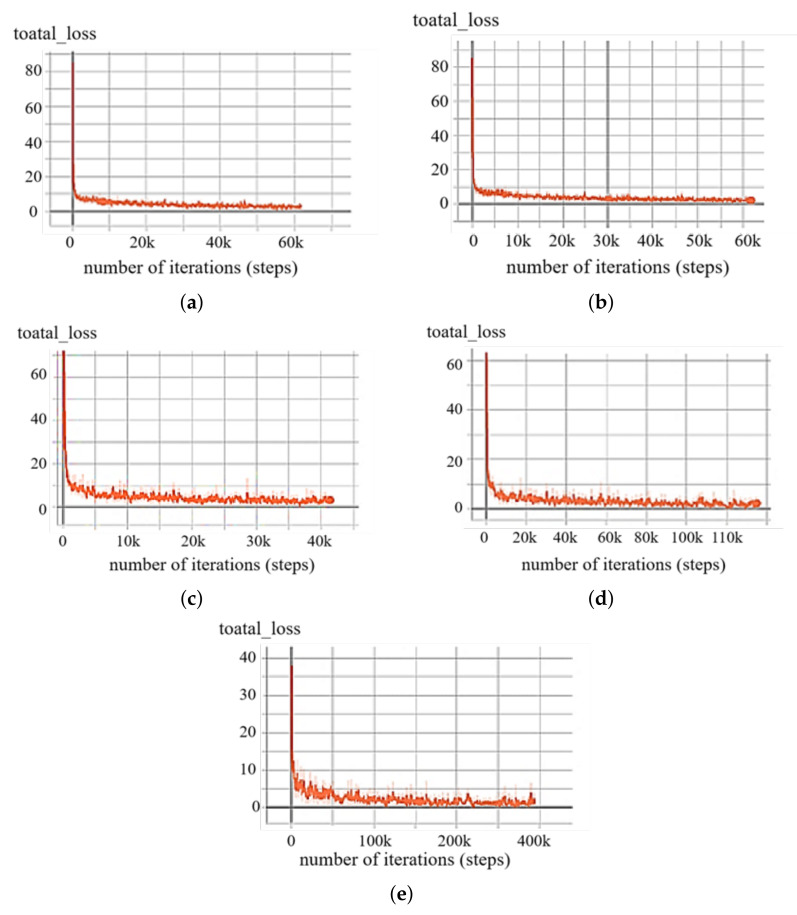
The results of the five algorithms’ experimental loss function: (**a**) YOLOv3; (**b**) improved network structure; (**c**) improved network structure + label smoothing; (**d**) improved network structure + cosine annealing learning rate; (**e**) comprehensively improved network.

The loss function curves of the five algorithms are shown in Figure 8. We can see that the loss values of the loss functions are gradually approaching 0. That is, the five models trained by the neural network are all stable.

The above objective evaluation indexes the accuracy, the mAP, the $F_{1}$ -score, and loss curve evaluate the five algorithms. The results show that the FPS values of the five algorithms are almost equal, and the loss curve converges to 0. However, the detection accuracy of the comprehensively improved algorithm in this paper is the highest among the five algorithms, so the overall effect is the best. It quantitatively shows the effectiveness of the comprehensively improved algorithm we proposed.

### 5.3. Visual Analysis of Detection Effect

(1) Image detection results

**Figure 9:**
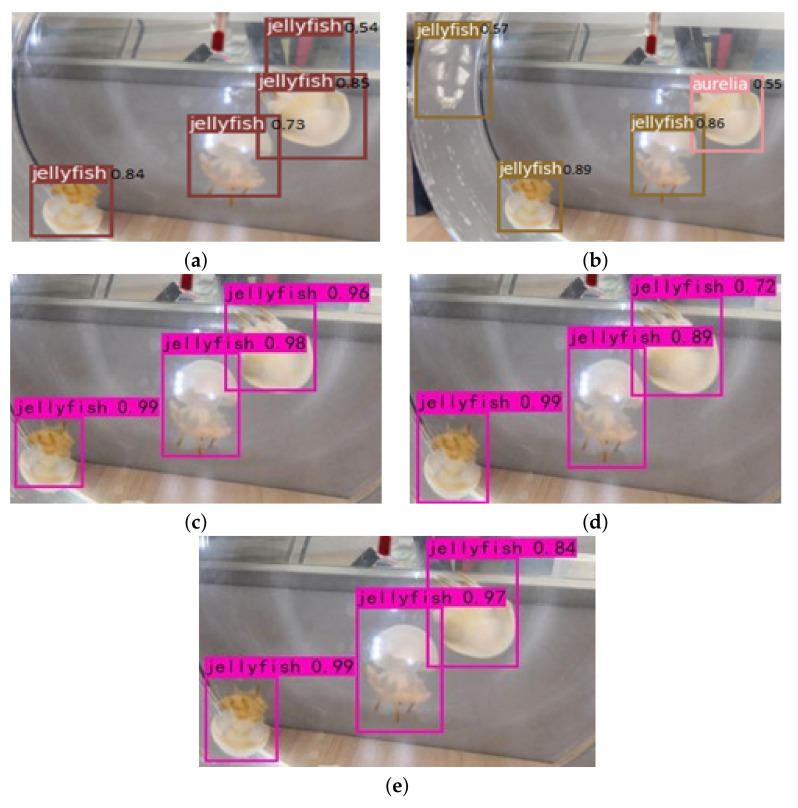
The detection results of jellyfish by the five algorithms: (**a**) YOLOv3; (**b**) improved network structure; (**c**) improved network structure + label smoothing; (**d**) improved network structure + cosine annealing learning rate; (**e**) comprehensively improved network.

**Figure 10:**
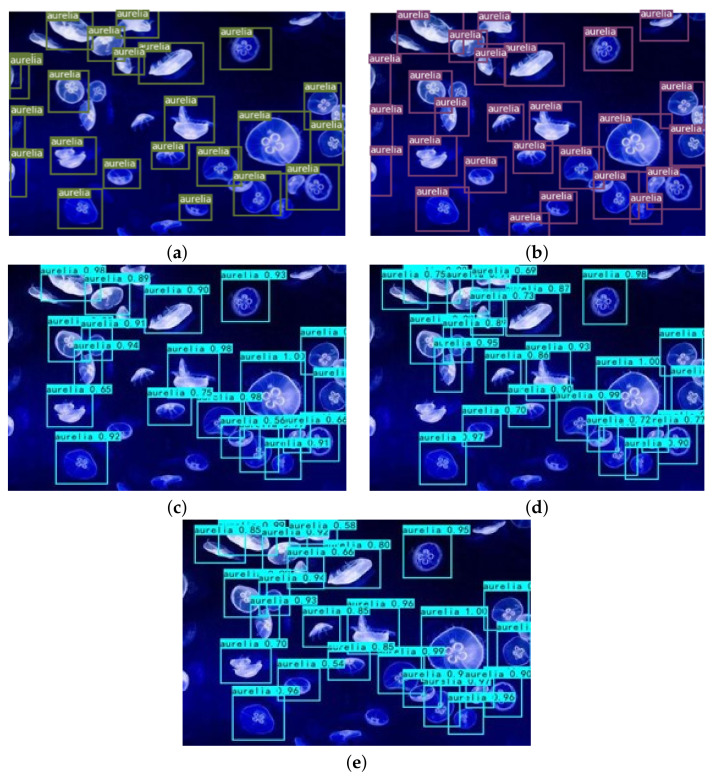
The detection results of *A. aurit* by five algorithms: (**a**) YOLOv3; (**b**) improved network structure; (**c**) improved network structure + label smoothing; (**d**) improved network structure + cosine annealing learning rate; (**e**) comprehensively improved network.

The comparison of detection results is an important step to verify the effectiveness of the algorithm. For this reason, several groups of visualization experiments have been conducted in this paper. The original jellyfish picture in Figure 9 comes from the network, which contains seven jellyfish. Two jellyfish are seriously blocked, and one jellyfish only appears half of the individual in the image. The original jellyfish picture in Figure 10 comes from the ImageNet data set, in which there are several *A. aurita*, especially some small individual *A. aurita*.

We can see from Figure 9a that the original YOLOv3 algorithm detects more than an *R. esculentu* in the upper right corner. It can be seen from Figure 9b that the improved network can accurately detect two *R. esculentu*, but a *R. esculentu* is mistakenly detected as an *A. aurit*. It can be seen from Figure 9c–e that using the improved network and introducing the label smoothing method, the improved network and introducing cosine annealing learning rate, as well as the comprehensively improved algorithm, the numbers of jellyfish are relatively accurate. Three jellyfish are completely detected, and individual jellyfish are framed. However, the comprehensively improved algorithm is more accurate than the other two in locating *R. esculentu* in the upper right corner. The umbrella cap of *R. esculentu* is completely framed. From the confidence level corresponding to the boxes, the confidence level of the three boxes are 0.99, 0.98, and 0.96. The comprehensively improved algorithm in this chapter is slightly lower than it, with confidence levels of 0.99, 0.97, and 0.84, respectively. However, the positioning effect of the comprehensively improved algorithm is better than that of the network structure and the label smoothing method. To sum up, the improved algorithm in this paper has the best detection effect on *R. esculentu*.

We can see from Figure 10 that numbers of *A. aurit* detected by the algorithm is increasing, which shows that the comprehensively improved algorithm has the best positioning effect and a high confidence value. That is, the comprehensively improved algorithm can improve the detection effect of *A. aurit* as a whole.

(2) Real-time video detection results

**Figure 11:**
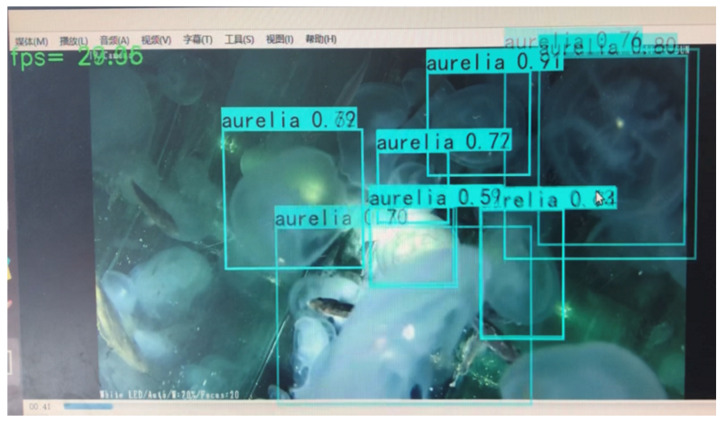
The video detection results of *A. aurit* by comprehensively improved algorithm.

Figure 11 is a screenshot of the jellyfish video detection process using the improved comprehensively improved algorithm in this paper. The video is collected in a large jellyfish culture tank in the aquarium, mainly containing *A. aurit*. The value in the upper left corner of the image is the FPS of this frame image by the comprehensively improved algorithm. It can be seen from the video screenshots that the comprehensively improved algorithm in this paper has high accuracy and good real-time performance in the jellyfish detection video, which can meet the requirements of real-time monitoring.

To sum up, the mAP, the $F_{1}$ -score, and the loss values of the improved algorithm in this paper are all the best, and the detection effect for jellyfish examples are also the best. Therefore, it can be shown that the comprehensively improved algorithm in this paper can improve the detection effect of jellyfish as a whole. That is, the accuracy is high. In addition, the FPS values of the algorithm adopted in this paper are 52.3 frames per second, which can meet the real-time requirements.

## 6. Conclusions

In this paper, aiming at the problem of low accuracy when the YOLOv3 algorithm is used in jellyfish detection, the feature extraction network, Darknet53, is improved on the basis of ensuring the real-time performance of the algorithm. A large-step cross-layer connection is introduced into the Darknet53 network to improve the feature extraction ability. The top-down FPN is added, and the information of the feature map is extracted repeatedly, thereby improving the detection accuracy. The learning rate method of label smoothing and cosine annealing is introduced in the training process to improve network accuracy. The experimental results show that the improved and optimized network structure improves the overall network feature extraction ability on the premise of ensuring the detection speed and can meet the requirements of jellyfish detection accuracy in real-time.

The jellyfish detection technology based on CNN is a relatively new research direction with a short development time. The system integrates optics, machine learning, and image processing theories and has a certain breadth and depth. In this paper, some algorithms have been studied. Due to the limited ability and energy, many aspects of the algorithm need to be further improved:

(1) In the jellyfish data set established in this paper, most images come from the network, while a small part of image comes from the laboratory’s collection, and the sample environment is single. The experimental background is to monitor the jellyfish situation in the Qinhuangdao Sea area, so it is necessary to collect more jellyfish images, which can effectively improve the practical application effect. In addition, when manually labeling data, we may make few wrongs labeling phenomena, which negatively influence model recognition. Therefore, we will collect the real sea environment for training to improve the practicability of the model.
(2) The algorithm is not currently applied in practice. The next step is to study and complete an integrated jellyfish detection platform or WeChat applet to monitor jellyfish in the ocean or provide tourists with convenient conditions to identify jellyfish quickly.
(3) Compared with YOLOv4, YOLOv3 was proposed a bit earlier, and it has more applications in target recognition detection. Therefore, our research group first completed the detection and recognition of jellyfish based on this network. Based on the above work foundation, the next step is to apply the YOLOv4, which is an improvement over YOLOv3, to the detection and recognition of jellyfish to enhance the performance of the model.

## Figures and Tables

**Table 1:** Data set distribution.

*C. purpurea*	*R. pulmo*	*P. camtschatica*	*A. okeni*	*A. aurita*	*P. punctata*	*R. esculentu*	Fish	Total
208	292	343	430	600	110	68	90	2141

**Table 2:** The setting of the training set, the verification set and the test set.

Dataset Settings	Numbers of Images
the training set	1713
the verification set	214
the test set	214
total	2141

**Table 3:** The results of evaluation parameters.

Image Quality Parameter	MSE	PSNR	SSIM	SNT
dark channel prior defogging	106.0336	51.8408	0.9919	7.4465
restrict adaptive histogram equalization	65.3919	64.1902	0.9968	7.4396
multi-scale retinal enhancement based on color recovery	**38.3273**	**78.0265**	**0.9989**	**7.5786**

**Table 4:** Network super parameter.

Parameter	YOLOv3	Improved Algorithm
learning rate	0.001	cosine annealing learning rate
loss function	category crossentropy	
opimtizer	Adam	
batch size	4	
epochs	training to model convergence	

**Table 5:** The accuracy results of the five algorithms.

Jellyfish Species	YOLOv3	Improved Network Structure	Improved Network Structure		Comprehensively Improved Algorithm
			Label Smoothing	Cosine Annealing Learning Rate	
*C. purpurea*	0.6835	0.8427	0.8627	0.8902	0.9245
*R. pulmo*	0.9699	0.9287	0.9812	0.9816	0.9878
*P. camtschatica*	0.9216	0.9526	0.9837	0.9981	0.9854
*A. okeni*	0.4950	0.7892	0.8213	0.8000	0.8439
*A. aurita*	0.9737	0.9376	0.9500	0.9278	0.9716
*P. punctata*	0.8457	0.9283	0.9196	0.9070	0.9672
*R. esculentu*	0.9569	0.9211	0.9086	0.8551	0.9624
fish	0.8235	0.9302	0.9708	0.9781	0.9998

**Table 6:** The comparison of the five algorithms’ mAP.

YOLOv3	Improved Network Structure	Improved Network Structure		Comprehensively Improved Algorithm
		Label Smoothing	Cosine Annealing Learning Rate	
0.8212	0.9038	0.9247	0.9172	**0.9553**

**Table 7:** The comparison of the five algorithms’ $F_{1}$ -score.

YOLOv3	Improved Network Structure	Improved Network Structure		Comprehensively Improved Algorithm
		Label Smoothing	Cosine Annealing Learning Rate	
0.8015	0.8430	0.8775	0.8600	**0.9137**

**Table 8:** The comparison of the five algorithms’ FPS.

YOLOv3	Improved Network Structure	Improved Network Structure		Comprehensively Improved Algorithm
		Label Smoothing	Cosine Annealing Learning Rate	
53.7	52.0	52.2	51.6	**52.3**

## Data Availability

Jellyfish dataset: Available online: https://github.com/srv/jf_object_detection (accessed on 30 August 2021).
